# Expert Consensus on the Diagnosis and Treatment of *NRG1/2* Gene Fusion Solid Tumors

**DOI:** 10.1055/s-0044-1781457

**Published:** 2024-02-27

**Authors:** Chunwei Xu, Qian Wang, Dong Wang, Wenxian Wang, Wenfeng Fang, Ziming Li, Aijun Liu, Jinpu Yu, Wenzhao Zhong, Zhijie Wang, Yongchang Zhang, Jingjing Liu, Shirong Zhang, Xiuyu Cai, Anwen Liu, Wen Li, Ping Zhan, Hongbing Liu, Tangfeng Lv, Liyun Miao, Lingfeng Min, Yu Chen, Jingping Yuan, Feng Wang, Zhansheng Jiang, Gen Lin, Long Huang, Xingxiang Pu, Rongbo Lin, Weifeng Liu, Chuangzhou Rao, Dongqing Lv, Zongyang Yu, Xiaoyan Li, Chuanhao Tang, Chengzhi Zhou, Junping Zhang, Junli Xue, Hui Guo, Qian Chu, Rui Meng, Jingxun Wu, Rui Zhang, Jin Zhou, Zhengfei Zhu, Yongheng Li, Hong Qiu, Fan Xia, Yuanyuan Lu, Xiaofeng Chen, Rui Ge, Enyong Dai, Yu Han, Weiwei Pan, Fei Pang, Qingqing He, Jintao Huang, Kai Wang, Fan Wu, Bingwei Xu, Liping Wang, Youcai Zhu, Li Lin, Yanru Xie, Xinqing Lin, Jing Cai, Ling Xu, Jisheng Li, Xiaodong Jiao, Kainan Li, Jia Wei, Huijing Feng, Lin Wang, Yingying Du, Wang Yao, Xuefei Shi, Xiaomin Niu, Dongmei Yuan, Yanwen Yao, Jianhui Huang, Yue Feng, Yinbin Zhang, Pingli Sun, Hong Wang, Mingxiang Ye, Zhaofeng Wang, Yue Hao, Zhen Wang, Bin Wan, Donglai Lv, Shengjie Yang, Jin Kang, Jiatao Zhang, Chao Zhang, Juanjuan Ou, Lin Shi, Yina Wang, Bihui Li, Zhang Zhang, Zhongwu Li, Zhefeng Liu, Nong Yang, Lin Wu, Huijuan Wang, Gu Jin, Guansong Wang, Jiandong Wang, Meiyu Fang, Yong Fang, Yuan Li, Xiaojia Wang, Yiping Zhang, Xixu Zhu, Yi Shen, Shenglin Ma, Biyun Wang, Lu Si, Yong Song, Yuanzhi Lu, Jing Chen, Zhengbo Song

**Affiliations:** 1 Department of Scientific Research, Institute of Cancer and Basic Medicine, Chinese Academy of Sciences, Hangzhou Zhejiang, People's Republic of China; 2 Department of Respiratory Medicine, Affiliated Jinling Hospital, Medical School of Nanjing University, Nanjing Jiangsu, People's Republic of China; 3 Department of Respiratory Medicine, Affiliated Hospital of Nanjing University of Chinese Medicine, Jiangsu Province Hospital of Chinese Medicine, Nanjing Jiangsu, People's Republic of China; 4 Department of Chemotherapy, Chinese Academy of Sciences University Cancer Hospital (Zhejiang Cancer Hospital), Hangzhou, Zhejiang, People's Republic of China; 5 Department of Medical Oncology, Sun Yat-sen University Cancer Center, State Key Laboratory of Oncology in South China, Collaborative Innovation Center for Cancer Medicine, Guangzhou Guangdong, People's Republic of China; 6 Department of Shanghai Lung Cancer Center, Shanghai Chest Hospital, Shanghai Jiao Tong University, Shanghai, People's Republic of China; 7 Senior Department of Pathology, the 7th Medical Center of PLA General Hospital, Beijing, People's Republic of China; 8 Department of Cancer Molecular Diagnostics Core, Tianjin Medical University Cancer Institute and Hospital, Tianjin, People's Republic of China; 9 Department of Guangdong Lung Cancer Institute, Guangdong Provincial Laboratory of Translational Medicine in Lung Cancer, Guangdong Provincial People's Hospital, Guangdong Academy of Medical Sciences, School of Medicine, Guangzhou Guangdong, People's Republic of China; 10 State Key Laboratory of Molecular Oncology, Department of Medical Oncology, National Cancer Center/National Clinical Research Center for Cancer/Cancer Hospital, Chinese Academy of Medical Sciences and Peking Union Medical College, Beijing, People's Republic of China; 11 Department of Medical Oncology, Lung Cancer and Gastrointestinal Unit, Hunan Cancer Hospital/The Affiliated Cancer Hospital of Xiangya School of Medicine, Central South University, Changsha Hunan, People's Republic of China; 12 Department of Thoracic Cancer, Jilin Cancer Hospital, Jilin Changchun, People's Republic of China; 13 Department of Translational Medicine Research Center, Key Laboratory of Clinical Cancer Pharmacology and Toxicology Research of Zhejiang Province, Affiliated Hangzhou First People's Hospital, Cancer Center, Zhejiang University School of Medicine, Hangzhou Zhejiang, People's Republic of China; 14 Department of VIP Inpatient, Sun Yat-Sen University Cancer Center, State Key Laboratory of Oncology in South China, Collaborative Innovation Center for Cancer Medicine, Guangzhou, Guangdong, People's Republic of China; 15 Department of Oncology, Second Affiliated Hospital of Nanchang University, Nanchang Jiangxi, People's Republic of China; 16 Key Laboratory of Respiratory Disease of Zhejiang Province, Department of Respiratory and Critical Care Medicine, Second Affiliated Hospital of Zhejiang University School of Medicine, Cancer Center, Zhejiang University, Hangzhou Zhejiang, People's Republic of China; 17 Department of Respiratory Medicine, Affiliated Drum Tower Hospital, Medical School of Nanjing University, Nanjing Jiangsu, People's Republic of China; 18 Department of Respiratory Medicine, Clinical Medical School of Yangzhou University, Subei People's Hospital of Jiangsu Province, Yangzhou Jiangsu, People's Republic of China; 19 Department of Medical Oncology, Fujian Medical University Cancer Hospital & Fujian Cancer Hospital, Fuzhou Fujian, People's Republic of China; 20 Department of Pathology, Renmin Hospital of Wuhan University, Wuhan Hubei, People's Republic of China; 21 Department of Internal Medicine, Cancer Center of PLA, Qinhuai Medical Area, Affiliated Jinling Hospital, Medical School of Nanjing University, Nanjing, Jiangsu, People's Republic of China; 22 Department of Integrative Oncology, Tianjin Medical University Cancer Institute and Hospital, Tianjin, People's Republic of China; 23 Department of Medical Oncology, Lung Cancer and Hunan Cancer Hospital/The Affiliated Cancer Hospital of Xiangya School of Medicine, Central South University, Changsha Hunan, People's Republic of China; 24 Department of Orthopaedic Oncology Surgery, Beijing Ji Shui Tan Hospital, Peking University, Beijing, People's Republic of China; 25 Department of Radiotherapy and Chemotherapy, Hwamei Hospital, University of Chinese Academy of Sciences, Ningbo Zhejiang, People's Republic of China; 26 Department of Pulmonary Medicine, Taizhou Hospital of Wenzhou Medical University, Taizhou Zhejiang, People's Republic of China; 27 Department of Respiratory Medicine, the 900th Hospital of the Joint Logistics Team (the Former Fuzhou General Hospital), Fujian Medical University, Fuzhou Fujian, People's Republic of China; 28 Department of Oncology, Beijing Tiantan Hospital, Capital Medical University, Beijing, People's Republic of China; 29 Department of Medical Oncology, Peking University International Hospital, Beijing, People's Republic of China; 30 Department of State Key Laboratory of Respiratory Disease, National Clinical Research Center for Respiratory Disease, Guangzhou Institute of Respiratory Health, The First Affiliated Hospital of Guangzhou Medical University (The First Affiliated Hospital of Guangzhou Medical University), Guangzhou Guangdong, People's Republic of China; 31 Department of Thoracic Oncology, Shanxi Academy of Medical Sciences, Shanxi Bethune Hospital, Taiyuan Shanxi, People's Republic of China; 32 Department of Oncology, Shanghai East Hospital, School of Medicine, Tongji University, Shanghai, People's Republic of China; 33 Department of Medical Oncology, The First Affiliated Hospital of Xi'an Jiaotong University, Xi'an Shaanxi, People's Republic of China; 34 Department of Oncology, Tongji Hospital of Tongji Medical College, Huazhong University of Science and Technology, Wuhan Hubei, People's Republic of China; 35 Department of Cancer Center, Union Hospital, Tongji Medical College, Huazhong University of Science and Technology, Wuhan Hubei, People's Republic of China; 36 Department of Medical Oncology, the First Affiliated Hospital of Medicine, Xiamen University, Xiamen Fujian, People's Republic of China; 37 Department of Medical Oncology, Cancer Hospital of China Medical University, Shenyang Liaoning, People's Republic of China; 38 Department of Medical Oncology, Sichuan Cancer Hospital & Institute, Sichuan Cancer Center, School of Medicine, University of Electronic Science and Technology, Chengdu Sichuan, People's Republic of China; 39 Department of Radiation Oncology, Fudan University Shanghai Cancer Center, Shanghai, People's Republic of China; 40 Key Laboratory of Carcinogenesis and Translational Research (Ministry of Education/Beijing), Department of Radiation Oncology, Peking University Cancer Hospital & Institute, Beijing, People's Republic of China; 41 Department of State Key Laboratory of Cancer Biology, National Clinical Research Center for Digestive Diseases and Xijing Hospital of Digestive Diseases, Fourth Military Medical University, Xi'an Shaanxi, People's Republic of China; 42 Department of Oncology, Jiangsu Province Hospital and Nanjing Medical University First Affiliated Hospital, Nanjing Jiangsu, People's Republic of China; 43 Department of General Surgery, Huadong Hospital Affiliated to Fudan University, Shanghai, People's Republic of China; 44 Department of Oncology and Hematology, China-Japan Union Hospital of Jilin University, Changchun Jilin, People's Republic of China; 45 Department of Gastrointestinal Oncology, Harbin Medical University Cancer Hospital, Harbin Heilongjiang, People's Republic of China; 46 Department of Cell Biology, College of Medicine, Jiaxing University, Jiaxing Zhejiang, People's Republic of China; 47 Department of Medical, Shanghai OrigiMed Co., Ltd., Shanghai, People's Republic of China; 48 Department of Medical, Stone Pharmaceuticals (Suzhou) Co., Ltd., Shanghai, People's Republic of China; 49 Department of Biotherapy, Cancer Institute, First Affiliated Hospital of China Medical University, Shenyang, People's Republic of China; 50 Department of Oncology, Baotou Cancer Hospital, Baotou Inner Mongolia, People's Republic of China; 51 Department of Thoracic Disease Diagnosis and Treatment Center, Zhejiang Rongjun Hospital, The Third Affiliated Hospital of Jiaxing University, Jiaxing Zhejiang, People's Republic of China; 52 Department of Oncology, Lishui Municipal Central Hospital, Lishui Zhejiang, People's Republic of China; 53 Department of Interventional Pulmonary Diseases, Anhui Chest Hospital, Hefei Anhui, People's Republic of China; 54 Department of Medical Oncology, Qilu Hospital, Cheeloo College of Medicine, Shandong University, Jinnan Shangdong, People's Republic of China; 55 Department of Medical Oncology, Shanghai Changzheng Hospital, Naval Medical University, Shanghai, People's Republic of China; 56 Department of Oncology, Shandong Provincial Third Hospital, Cheeloo College of Medicine, Shandong University, Jinan Shandong, People's Republic of China; 57 Department of the Comprehensive Cancer Center, Affiliated Drum Tower Hospital, Medical School of Nanjing University, Nanjing Jiangsu, People's Republic of China; 58 Department of Pathology, Shanxi Academy of Medical Sciences, Shanxi Bethune Hospital, Taiyuan Shanxi, People's Republic of China; 59 Department of Oncology, The First Affiliated Hospital of Anhui Medical University, Hefei Anhui, People's Republic of China; 60 Department of Interventional Oncology, The First Affiliated Hospital, Sun Yat-sen University, Guangzhou Guangdong, People's Republic of China; 61 Department of Respiratory Medicine, Huzhou Hospital, Zhejiang University School of Medicine, Huzhou Zhejiang, People's Republic of China; 62 Department of Gynecologic Radiation Oncology, Chinese Academy of Sciences University Cancer Hospital (Zhejiang Cancer Hospital), Hangzhou, Zhejiang, People's Republic of China; 63 Department of Oncology, the Second Affiliated Hospital of Medical College, Xi′an Jiaotong University, Xi'an Shaanxi, People's Republic of China; 64 Department of Pathology, The Second Hospital of Jilin University, Changchun Jilin, People's Republic of China; 65 Senior Department of Oncology, The 5th Medical Center of PLA General Hospital, Beijing, People's Republic of China; 66 Department of Radiation Oncology, Affiliated Jinling Hospital, Medical School of Nanjing University, Nanjing, Jiangsu, People's Republic of China; 67 Department of Respiratory Medicine, The Affiliated Jiangning Hospital of Nanjing Medical University, Nanjing Jiangsu, People's Republic of China; 68 Department of Clinical Oncology, The 901 Hospital of Joint Logistics Support Force of People Liberation Army, Hefei Anhui, People's Republic of China; 69 Department of Thoracic Surgery, Chuxiong Yi Autonomous Prefecture People's Hospital, Chuxiong, People's Republic of China; 70 Department of Oncology and Southwest Cancer Center, Southwest Hospital, Third Military Medical University (Army Medical University), Chongqing, People's Republic of China; 71 Department of Respiratory Medicine, Zhongshan Hospital, Fudan University, Shanghai, People's Republic of China; 72 Department of Oncology, The First Affiliated Hospital, College of Medicine, Zhejiang University, Hangzhou Zhejiang, People's Republic of China; 73 Department of Oncology, The Second Affiliated Hospital of Guilin Medical University, Guilin Guangxi, People's Republic of China; 74 Department of International Cooperative Laboratory of Traditional Chinese Medicine Modernization and Innovative Drug Discovery of Chinese Ministry of Education (MOE), Guangzhou City Key Laboratory of Precision Chemical Drug Development, School of Pharmacy, Jinan University, Guangzhou, Guangdong, People's Republic of China; 75 Key Laboratory of Carcinogenesis and Translational Research (Ministry of Education/Beijing), Department of Pathology, Peking University Cancer Hospital & Institute, Beijing, People's Republic of China; 76 Department of Internal Medicine, The Affiliated Cancer Hospital of Zhengzhou University, Henan Cancer Hospital, Zhengzhou Henan, People's Republic of China; 77 Department of Bone and Soft-tissue Surgery, Chinese Academy of Sciences University Cancer Hospital (Zhejiang Cancer Hospital), Hangzhou Zhejiang, People's Republic of China; 78 Institute of Respiratory Diseases, Xinqiao Hospital, Third Military Medical University, Chongqing, People's Republic of China; 79 Department of Pathology, Affiliated Jinling Hospital, Medical School of Nanjing University, Nanjing, Jiangsu, People's Republic of China; 80 Department of Medical Oncology, Sir Run Run Shaw Hospital, Zhejiang University, Hangzhou Zhejiang, People's Republic of China; 81 Department of Pathology, Fudan University Shanghai Cancer Center, Shanghai, People's Republic of China; 82 Department of Thoracic Surgery, Affiliated Jinling Hospital, Medical School of Nanjing University, Nanjing Jiangsu, People's Republic of China; 83 Department of Oncology, Key Laboratory of Clinical Cancer Pharmacology and Toxicology Research of Zhejiang Province, Affiliated Hangzhou Cancer Hospital, Cancer Center, Zhejiang University School of Medicine, Hangzhou Zhejiang, People's Republic of China; 84 Department of Breast Cancer and Urological Medical Oncology, Fudan University Shanghai Cancer Center, Department of Oncology, Shanghai Medical College, Fudan University, Shanghai, People's Republic of China; 85 Key Laboratory of Carcinogenesis and Translational Research (Ministry of Education/Beijing), Department of Melanoma and Sarcoma, Peking University Cancer Hospital & Institute, Beijing, People's Republic of China; 86 Department of Clinical Pathology, the First Affiliated Hospital of Jinan University, Guangzhou Guangdong, People's Republic of China

**Keywords:** tyrosine receptor kinase, monoclonal antibodies, precision medicine, targeted therapy, solid tumor, fusion

## Abstract

The fusion genes *NRG1* and *NRG2* , members of the epidermal growth factor (EGF) receptor family, have emerged as key drivers in cancer. Upon fusion, *NRG1* retains its EGF-like active domain, binds to the ERBB ligand family, and triggers intracellular signaling cascades, promoting uncontrolled cell proliferation. The incidence of *NRG1* gene fusion varies across cancer types, with lung cancer being the most prevalent at 0.19 to 0.27%. CD74 and SLC3A2 are the most frequently observed fusion partners. RNA-based next-generation sequencing is the primary method for detecting *NRG1* and *NRG2* gene fusions, whereas pERBB3 immunohistochemistry can serve as a rapid prescreening tool for identifying *NRG1* -positive patients. Currently, there are no approved targeted drugs for *NRG1* and *NRG2* . Common treatment approaches involve pan-ERBB inhibitors, small molecule inhibitors targeting ERBB2 or ERBB3, and monoclonal antibodies. Given the current landscape of *NRG1* and *NRG2* in solid tumors, a consensus among diagnostic and treatment experts is proposed, and clinical trials hold promise for benefiting more patients with *NRG1* and *NRG2* gene fusion solid tumors.

## Introduction

Gene fusion caused by chromosomal rearrangement is a common event in solid tumors, driving tumorigenesis. The identification and targeting of fusion genes have been significant breakthroughs in medicine. Chromosomal rearrangements of receptor tyrosine kinases (RTKs) can generate oncogenic fusion protein kinases. Several tyrosine kinase inhibitors (TKIs) have been approved for treating solid malignancies with RTK fusions. 1 The epidermal growth factor (EGF) receptor family belongs to the type I RTK family. *NRG1* and *NRG2* genes encode neuroregulin 1 and 2 proteins, respectively, which are part of the EGF ligand family. *NRG1* gene fusion activates and retains the EGF-like domain of the NRG1 protein, continuously binding to ERBB receptor family members (ERBB2 and ERBB4). This initiates intracellular signaling cascades, leading to sustained cell proliferation and tumorigenesis. 2

Although *NRG1* gene fusion in solid tumors is rare (0.2%), patients with *NRG1* fusion tumors often have a poor response to standard treatments. Disrupting *NRG1* binding to ERBB3 or impacting ERBB2/ERBB3 heterodimerization can reduce the volume of *NRG1* fusion tumors in various solid tumors. 3 *NRG1* is an emerging oncogenic driver and a potential therapeutic target, but no approved targeted drugs are available for *NRG1* fusion tumors. *NRG2* fusion has also been found in lung adenocarcinoma patients, but further understanding of its biological functions is needed. 4 5

This article summarizes the biological behaviors of NRG1 and NRG2 fusion-related proteins and introduces molecular characteristic data of *NRG1* gene fusion in solid tumors from the largest-scale database. It proposes a screening strategy for *NRG1/2* gene fusion solid tumors based on existing domestic resources. Ongoing clinical trials targeting *NRG1* fusion solid tumors are also summarized, along with proposed treatment consensus.

## The Biological Basis of the *NRG1/2* Gene

### The Gene Structures and Biological Functions of the *NRG1/2* Gene

RTKs are essential in drug development, with the ERBB family, including ERBB1 (EGFR), ERBB2 (HER2), ERBB3 (HER3), and ERBB4 (HER4), being transmembrane RTKs known as the EGF receptor family. The tyrosine kinase ligand family, which includes the neuregulin family (NRGs), consists of six protein isoforms: NRG1, NRG2, NRG3, NRG4, NRG5 (tomoregulin), and NRG6 (neuroglycan C). These ligands all contain an extracellular EGF-like domain that activates the ERBB RTK. They are crucial for the development of the nervous and cardiovascular systems. 6 7

#### *NRG1*

**Fig. 1:**
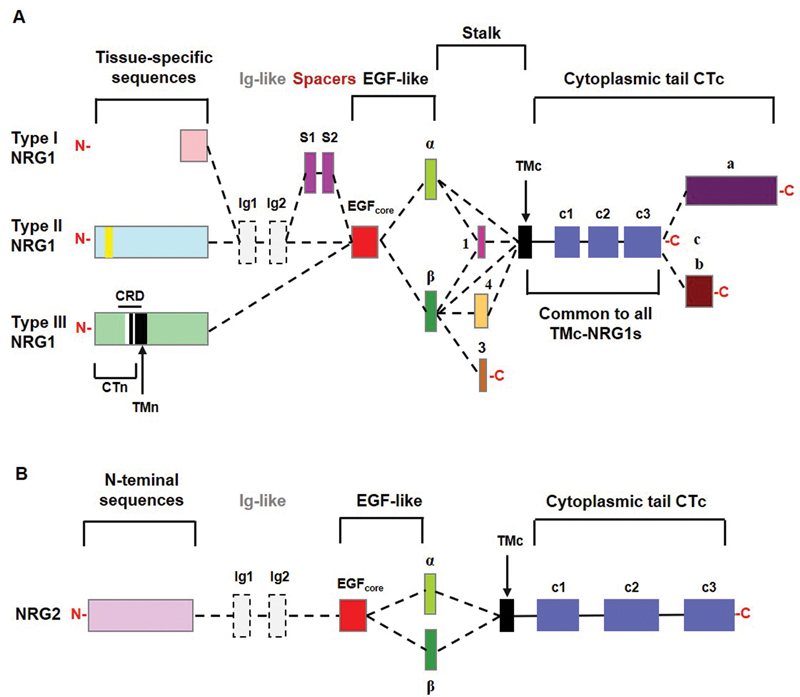
*NRG1* and *NRG2* structures. ( **A** ) *NRG1* possesses I, II, and III subtype structures patterns. The coding sequences of the same isoform vary due to diverse transcription start sites and alternative splicing of *NRG1* gene promoters. It is worth noting that the EGF-like domain alone has the capability to efficiently activate homologous ERBB receptor tyrosine kinases. N and C marked in red represent the N-terminal and C-terminal of NRG1 protein, respectively. To obtain further information, please refer to the relevant literature. 2 38 ( **B** ) NRG2 structure. CRD, cysteine-rich domain; CTc, cytoplasmic tail domain C terminal of the EGF-like domain; TMc, transmembrane domain C terminal of the EGF-like domain; TMn, transmembrane domain N-terminal of the EGF-like domain.

The *NRG1* gene, also known as Neuregulin 1, Heregulin, Neu differentiation factor, Glial growth factor, and Acetylcholine receptor-inducing activity, is located at 8p21. 8 9 10 11 12 *NRG1* interacts with ERBB3 and ERBB4 through its EGF-like domain, tissue specificity, and immunoglobulin-like domain. 13 *NRG1* has multiple isoforms and structural differences, with six protein subtypes (I–VI) and at least 31 gene subtypes. The NRG1 protein consists of the EGF-like domain, the N-terminal sequence (type I, II, or III), and the C-terminal sequence (transmembrane or not). Type I and II NRGs are also referred to as “Ig-NRGs,” whereas type III NRGs are known as “CRD-NRGs.” The fusion-involved subtype of NRG1 belongs to type III and has a higher affinity for receptor binding than the α-type. This difference in binding affinity contributes to the oncogenic properties of NRG1 IIIβ compared with NRG1 IIIα. NRG1 is initially produced as a membrane-anchored precursor, and proteolysis releases the EGF-like domain, activating ERBB3 and ERBB4. The interaction between NRG1 and ERBB3 can lead to heterodimerization, particularly with ERBB2, facilitating downstream signaling pathways such as PI3K/AKT and MAPK. *NRG1* can also interact with ERBB4, forming homodimers or heterodimers with ERBB2/ERBB3, further activating multiple pathways 14 15 ( Fig. 1A ).

#### *NRG2*

The *NRG2* gene, also known as Divergent of neuregulin 1, Neural and thymus derived activator for ErbB kinases, and Neuregulin 2, is located at 5q13.2. 16 17 18 NRG2 has two isoforms, α and β, due to different splicing sites. Research has shown that NRG2β is a high-affinity ligand for ERBB4, strongly stimulating ERBB4 tyrosine phosphorylation. On the other hand, the splicing isoform NRG2α is a low-affinity ligand for ERBB4 and does not strongly stimulate ERBB4 phosphorylation 19 ( Fig. 1B ).

### Fusion and Carcinogenic Mechanism of *NRG1/2*

The activation or overexpression of NRGs has been shown to regulate tumor cell growth, invasion, and angiogenesis. These genes are associated with various types of tumors including breast cancer, ovarian cancer, endometrial cancer, colorectal cancer, gastric cancer, lung cancer, thyroid cancer, glioma, medulloblastoma, melanoma, and head and neck squamous cell carcinoma. 8 20 21 In solid tumors, gene fusion is a significant driver mutation. Specifically, *NRG1* gene fusion is considered a potential targetable oncogenic driver. The oncogenicity of *NRG1* and *NRG2* gene fusions relies on maintaining an intact EGF-like domain without frameshift mutations. 2 Knockout mouse models with disrupted EGF-like domain (neuregulin ^δEGF-LacZ^ ) have demonstrated that all *NRG1* subtypes lose their function, leading to embryonic death due to cardiac and nervous system malformations. 22

The discovery of *NRG1* fusion dates back to 1997 in the breast cancer cell line MDA-MB-175, where it was identified as a tumor-specific DOC4-NRG1 transcript that promotes tumor cell proliferation. 23 In lung cancer, *NRG1* gene fusion results in the overexpression of the EGF-like domain of NRG1 on the cell surface. This enhances its binding ability with ERBB3, promoting heterodimerization of ERBB2/ERBB3 and subsequently activating downstream PI3K/AKT and MAPK signaling pathways. 24 Studies using *CD74* - *NRG1* transgenic mouse models have shown that the proliferation of *CD74* - *NRG1* cells is carcinogenic and accompanied by increased protein transcription levels of ERBB2 and ERBB3, indicating that *NRG1* gene fusion drives tumor development. 25 *NRG1* gene fusion is the first potential therapeutic oncogenic driver mutation specifically associated with a subtype of lung adenocarcinoma and is predominantly found in nonsmoking patients, in contrast to the tobacco-associated KRAS gene mutation. 24 In a transcriptome sequencing study of 25 never-smoking lung adenocarcinoma patients, one case of *CD74* - *NRG1* gene fusion was identified in a patient with invasive mucinous subtype. Mechanistically, CD74- *NRG1* gene fusion leads to extracellular expression of the EGF-like domain of NRG1 III-β3, providing a ligand for the ERBB2–ERBB3 receptor complex. Consequently, ERBB2 and ERBB3 are highly expressed in index cases, and phosphorylated ERBB3 is specifically expressed in fusion tumors ( *p* < 0.0001). In lung cancer cell lines expressing ERBB2 and ERBB3, ectopic expression of *CD74* - *NRG1* activates the ERBB3 and PI3K-AKT pathways, resulting in increased colony formation in soft agar. 26

Breakpoints on the *NRG1* chromosome were discovered by Adélaïde et al in two pancreatic cancer cell lines (PaTu I, SUIT-2), indicating that *NRG1* breakpoints may be a recurring phenomenon in solid tumors. 27 Subsequent studies on breast cancer, pancreatic cancer, and lung cancer tumor samples further emphasized the role of *NRG1* rearrangements in tumor development. 28 Comprehensive molecular detection techniques have revealed *NRG1* fusions in various other tumors, particularly in invasive mucinous lung adenocarcinoma (IMA) and KRAS wild type pancreatic ductal adenocarcinoma. 29 30 31 32 The identification of recurrent and potentially targetable *NRG1* fusions provides therapeutic opportunities for these tumors.

In addition to *NRG1* gene fusion, *CD74-NRG2* gene fusion has been detected in lung adenocarcinoma patients. NRG2 has moderate affinity with ERBB2/4 heterodimers, and phosphorylation of ERBB2/3/4 may serve as an alternative biomarker for pathway activation. 33 Immunohistochemical analysis of CD74-NRG2 samples showed moderate phosphorylation of ERBB4 in positive tumor cells, whereas EGFR, ERBB2, and ERBB3 did not show phosphorylation. On the other hand, ERBB family members were phosphorylated in *NRG1* fusion tumor cells, suggesting that ERBB4 inhibitors may be effective drugs for *NRG2* gene fusion tumors. 4

## Epidemiology of *NRG1/2* Gene Fusion in Solid Tumors

### Mutation Frequency of *NRG1/2* Fusion

The occurrence rate of *NRG1* and *NRG2* gene fusion in solid tumors is extremely rare. The overall mutation frequency of *NRG1* gene fusion in all solid tumors is approximately 0.2%, but in certain patient subgroups, the mutation frequency can be as high as 30%. A study in the United States found an occurrence rate of *NRG1* gene fusion of 0.19% among 21,858 cases of solid tumors. The most common tumor types with *NRG1* gene fusion are gallbladder cancer, pancreatic cancer, renal cell carcinoma, ovarian cancer, nonsmall cell lung cancer (NSCLC), breast cancer, sarcoma, and bladder cancer. The incidence rates of other tumor types are all less than 0.1%. 3 Data from a population of solid tumor patients in Korea showed an occurrence rate of *NRG1* gene fusion of 0.27%, with lung cancer being the most common tumor type. 34 Another study based on data from 13,089 cases of NSCLC in China showed an occurrence rate of *NRG1* gene fusion of 0.19%. 35 IMA accounts for approximately 57 to 61% of *NRG1* fusion NSCLC and slightly more than half of *NRG1* fusion NSCLC patients have never smoked. 36 37

The breakpoints of *NRG1* fusion are typically found in three specific intronic regions: (1) a 47-kb region between exon 1 and exon 2; (2) a 955-kb region between exon II and exon 2; (3) a region between exon 5 and exon 6, including exon III, with a length of 111 kb. 36 The occurrence rate of NRG2 fusion is even rarer, with a frequency 5 to 10 times lower than that of *NRG1* . 4 5 38

### Fusion Partners of *NRG1* Gene Fusion

*NRG1* gene fusion can have different partners, which affects the biological properties of the synthesized chimeric protein. The NRG1 protein has a domain similar to EGF and acts as a ligand for ERBB3. The ligand can be localized in the complex, while the partner provides a transmembrane domain that binds the ligand to the membrane. In most cases, the partner facilitates the interaction between the ligand and the ERBB3 protein on adjacent cells. CD74 and SLC3A2 are the most common upstream fusion partners, but other partner genes include ATP1B1, CDH1, CLU, CRADD, FUT10, INCENP, KIF22, RBPMS, SLC20A2, VWA8, and XKR6, among others. 34

### Other Molecular Characteristics of *NRG1* Gene Fusion

Multiple studies have consistently shown that *NRG1* gene fusions are generally mutually exclusive with driver genes such as EGFR, ALK, and ROS1. This indicates that *NRG1* gene fusion may act as a strong driver mutation promoting the occurrence and development of tumors. Co-occurring mutations with *NRG1* gene fusions include TP53 (54.5%), KRAS, BRAF, PIK3CA, NF1, and NF2, among others. 3 34 Among 15 patients with solid tumors harboring *NRG1* gene fusions, the median tumor mutation burden was 3.9/Mb (range: 1.0–51.20/Mb), and the median microsatellite instability was 1.98% (range: 1.0–5.0%). 34

We believe that *NRG1* and *NRG2* gene fusions are rare but important targetable oncogenic alterations. Ideally, all advanced and metastatic solid tumors should be systematically tested for *NRG1* and *NRG2* gene fusions, along with other actionable oncogenic drivers. Molecular testing should be performed at the time of diagnosis, especially for patients with a histopathological diagnosis of IMA. Considering the frequent breakpoints in the intronic region of the *NRG1* gene, it is crucial to include intronic coverage when selecting the testing method, especially gene sequencing.

## Detection of *NRG1/2* Fusion

Chromosomal translocation is the primary cause of fusion genes, and accurate diagnosis of fusion genes is essential for effective treatment. In the clinical translation of NRG fusion α and β subtypes, it is crucial to avoid false negatives and minimize the need for further confirmation testing due to the diversity and rarity of NRG fusion variants. This requires advanced testing technology with high sensitivity. The standardization of operating procedures can improve the accuracy of detection. 38 Additionally, considering the limited availability of resources in many countries, cost-effectiveness is also an important factor to consider in the testing method. To optimize screening, specific tumor samples and knowledge of NRG fusions in specific cancer types should be combined. Combining multiple testing methods can further enhance the accuracy and reliability of *NRG1* fusion detection.

### Immunohistochemistry

Immunohistochemistry (IHC) can indirectly detect the fusion status of *NRG1* and *NRG2* by detecting the protein expression levels of *NRG1* or *NRG2* and their fusion partners in tumor tissues. IHC has advantages such as fast turnaround time, low cost, high sensitivity, and strong specificity. It relies on specific antibodies that can identify fusion proteins in tumor tissues. However, the selection of antibodies can significantly impact the results, and not all fusion variants may be detectable by specific antibodies.

Indirect detection of pERBB3 immunostaining may serve as a powerful predictive marker for *NRG1* fusion, as *NRG1* fusion can lead to increased fusion products and chimeric ligands, resulting in ERBB2/ERBB3 heterodimerization and phosphorylation-mediated activation of the ERBB3 receptor. 26 In a study cohort of 85 Caucasian patients, *NRG1* rearrangements were investigated in 51 IMA patients and 34 non-IMA patients using *NRG1* fluorescence in situ hybridization (FISH), pERBB3 immunohistochemistry, and RNA target sequencing. The findings revealed that 31% of IMA and 3% of non-IMA patients had *NRG1* gene rearrangements, indicating that pERBB3 immunohistochemistry had a sensitivity of 94% and specificity of 100% in the 51 IMA samples, as well as a sensitivity of 100% and specificity of 94% in the 34 non-IMA adenocarcinoma samples. Additionally, CD74-NRG1 fusion transcripts were detected in 4 *NRG1* -positive IMA patients. Importantly, all IMA cases with abnormal pERBB3 expression exhibited *NRG1* gene rearrangement. 39 Furthermore, in a study involving 245 lung adenocarcinoma samples, pERBB3 immunohistochemical detection demonstrated a sensitivity of 100% and specificity of 97.5%. 26 Thus, pERBB3 immunohistochemical detection may serve as a rapid and effective prescreening method for identifying *NRG1* -positive patients.

### Fluorescence in Situ Hybridization

FISH is a widely utilized method for visualizing and confirming the presence of *NRG1* and *NRG2* fusions in paraffin-embedded tissue samples. This technique employs fluorescently labeled probes that specifically bind to the fusion genes, enabling precise localization and assessment of fusion events. FISH is particularly valuable in identifying the specific fusion partners and breakpoints involved. When there is a suspicion of *NRG1* or *NRG2* fusion with distinct characteristics, FISH can be employed for genotyping purposes. Break-apart FISH, a commonly employed clinical method and one of the Food and Drug Administration (FDA)-approved techniques for detecting ALK rearrangements, detects gene fusions. However, unlike ALK fusion FISH testing, the scoring criteria for determining *NRG1* fusion positivity lack comprehensive study and validation. Consequently, the current criteria for *NRG1* FISH testing positivity temporarily adopt the 15% separation signal threshold used in ALK testing, pending favorable validation data for widespread adoption of *NRG1* FISH. 38 While FISH testing has demonstrated success in NSCLC, 40 it was unable to detect *NRG1* fusions in two out of three cases of KRAS wild type pancreatic ductal adenocarcinoma with complex *NRG1* rearrangement patterns. 31 In addition to its inability to detect complex rearrangement patterns, FISH has other limitations, such as the restricted ability to simultaneously test multiple targets and the inability to determine if fusion partners express fusion products or if other co-mutations are present. Therefore, due to its high cost, low sensitivity, and specificity, we do not recommend FISH as a routine screening method for *NRG1* fusion detection.

### RNA-Based Next-Generation Sequencing

Transcriptome sequencing using second-generation sequencing technology enables accurate identification of *NRG1* and *NRG2* fusions by comparing gene expression profiles between tumor and normal tissues. This method provides comprehensive information about fusion transcripts and can detect new fusion events. RNA-based next-generation sequencing (NGS) is the optimal tool for discovering fusion genes at the transcriptional level due to the chimeric nature of fusion transcripts. The frequency of *NRG1* or *NRG2* fusions can be calculated using the number of connected reads, including the β/α isoform ratio. However, RNA-based NGS has limitations in obtaining sufficient quality and quantity of RNA from clinical samples, especially formalin-fixed paraffin-embedded tissues. In the eNRGy1 clinical trial, a combination of DNA and/or RNA NGS and FISH was employed to identify *NRG1* fusions. The detection rate of *NRG1* fusion using RNA-based NGS was found to be 74% (81/110), whereas the detection rate using DNA-based NGS was only 26%. This highlights the superior advantages of RNA-based NGS in fusion detection. 37

#### Whole Transcriptome Sequencing

Whole transcriptome sequencing (WTS) is the most comprehensive method for detecting gene fusions, particularly in identifying new fusion partners. WTS directly sequences transcribed mRNA without relying on initial adapter ligation steps. 30 31 40 41 Unlike targeted RNA sequencing, WTS does not require prior knowledge of fusion partners. However, WTS has limitations such as high requirements for sample quality and quantity, complex data analysis, high cost, and difficulty in detecting low-frequency events.

#### Targeted RNA-Sequencing Panel

Targeted RNA sequencing technology, such as anchored multiplex polymerase chain reaction (AMP), evaluates specific gene expression, mutations, and fusions and improves sequencing coverage by analyzing multiple genes in a single assay. 42 43 AMP is commercially available but mainly targets genes like ALK, RET, and ROS1 and covers the *NRG1* gene. 44 However, it cannot reliably detect *NRG2* gene fusions due to the lack of specific primers for *NRG2* gene amplification, which is a disadvantage compared with WTS. 3 38

### DNA-Based Next-Generation Sequencing

DNA-based NGS technology is widely used for tumor and plasma gene typing. It is a high-throughput sequencing method that provides comprehensive genetic information with reduced costs and time. Hybrid capture technology, a commonly used method, enables the sequencing of translocation breakpoints. DNA-NGS technology can identify most *NRG1* gene fusions and determine their breakpoints. However, it may miss fusions with large introns and cannot determine fusion protein functionality. Therefore, we recommend using a DNA gene testing panel that covers the intronic regions of *NRG1* and *NRG2* genes.

### Reverse Transcription-Polymerase Chain Reaction

Reverse transcription-polymerase chain reaction is a reliable method for detecting fusion transcripts of *NRG1* and *NRG2* genes. It involves reverse transcription of RNA into cDNA, followed by amplification using fusion gene-specific primers. This method accurately detects fusion breakpoints and is commonly used for validation, especially for partner genes with a high fusion breakpoint occurrence rate. However, it is not suitable for identifying new fusion partners and may not be sensitive enough for low-abundance fusion transcripts. 45 Therefore, it is not included in our recommended screening strategy.

### Screening Recommendations for *NRG1/2* Fusion

Despite advancements in detection methods, challenges remain in identifying *NRG1* and *NRG2* gene fusions. These include difficulties in detecting low-abundance fusion transcripts, the need for high-quality samples, lack of standardized methods, and low sensitivity for rare fusion events in heterogeneous tumors.

**Fig. 2:**
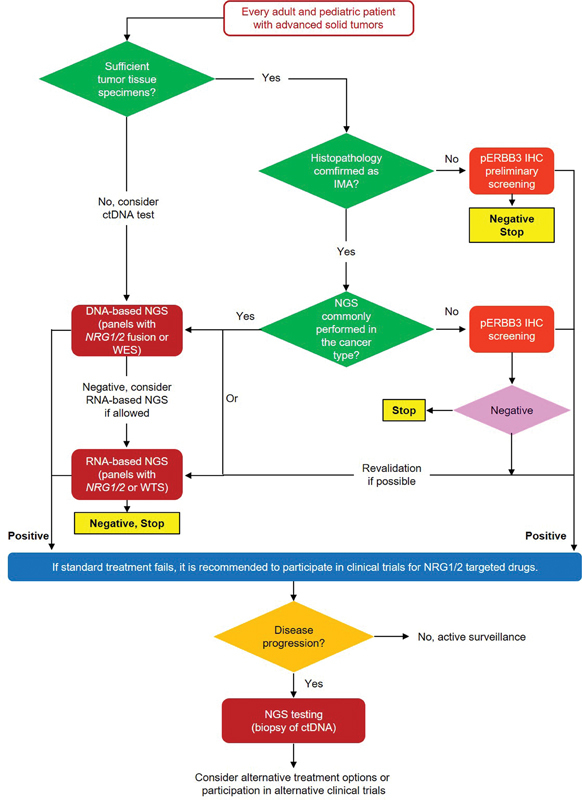
The recommended procedure for the diagnosis and treatment of *NRG1/2* gene fusion solid tumors. CTC, circulating tumor cells; IHC, immunohistochemistry; IMA, invasive mucinous adenocarcinoma; NGS, next-generation sequencing; WES, whole exome sequencing; WTS, whole transcriptome sequencing.

To enhance the identification of *NRG1* gene fusion solid tumor patients, we recommend using DNA or RNA NGS panels targeting the intronic regions of *NRG1/2* , or pERBB3 immunohistochemistry as the primary screening strategy. RNA NGS technology is particularly recommended when histology and molecular subtypes are unclear. Specific detection strategies and workflow information were listed as follow ( Table 1 ; Fig. 2 ).

**Table 1:** Consensus on the diagnosis and treatment of *NRG1/2* gene fusion solid tumors

	Consensus no.	Key points	Recommendation level
Detection time point	Consensus 1	A *NRG1/2* gene fusion test, in parallel to other actionable oncogenic drivers' tests is recommended for every adult and pediatric patient with advanced or metastatic solid tumor at diagnosis. NGS testing contain *NRG1/2* gene fusions is strongly recommended for invasive mucinous lung adenocarcinoma confirmed by histopathology	Strongly recommended
	Consensus 2	Advance or metastatic adult and pediatric solid tumor patients should consider *NRG1/2* gene fusion testing before or during standard treatment (recommended). For locally advanced invasive mucinous lung adenocarcinoma patients with high incidence of *NRG1/2* gene fusion, a *NRG1/2* gene fusion testing is strongly recommended before neoadjuvant therapy	Strongly recommended
Detection method	Consensus 3	Preferred tumor histological specimens should be used for fusion gene testing. If sufficient tumor histological specimens cannot be obtained, cytological specimens may be selected. Prior to fusion gene testing, tissue or cytological specimens should be evaluated for tumor cell content by professional pathologists. If sufficient tumor histological or cytological specimens cannot be obtained, liquid biopsy is recommended as a supplementary testing method	Strongly recommended
	Consensus 4	The main methods for *NRG1/2* gene fusion testing are whole transcriptome sequencing (WTS), RNA-based NGS panels, and DNA-based NGS panels covering the intronic regions of *NRG1/2* . The selection of testing platforms and methods should be made reasonably based on sample type, tumor cell content, specimen quality, platform accessibility, testing turnaround time, and cost. RNA-based NGS panels have higher sensitivity than DNA-based NGS panels. If necessary, multiple platforms can be used for complementation and verification, especially when IHC results are positive and DNA-based NGS panel results are negative. In such cases, it is strongly recommended to use the third detection method, RNA-based NGS panel, for confirmation	Strongly recommended
	Consensus 5	pERBB3 immunohistochemical test may serve as a rapid and effective prescreening method for identifying *NRG1* fusion patients	Strongly recommended
Detection strategy	Consensus 6	Each hospital should establish standardized procedures for *NRG1/2* gene fusion testing. Our expert group will regularly issue recommendations on the importance of NGS testing for each type of cancer. Given the rapid development of precision medicine for tumors, promoting the development of precision medicine at different levels of hospitals with *NRG1/2* gene fusion testing as a benchmark is of great significance	Recommended
Detection quality control	Consensus 7	All testing should be conducted in accredited laboratories. It is recommended to select laboratories accredited by authoritative institutions such as ISO15189, CAP, and CLIA for testing. The laboratory should perform internal and external quality control related to *NRG1/2* gene fusion testing in accordance with relevant regulations	Strongly recommended
	Consensus 8	In addition to basic information and quality control data, the testing report should also include tumor cell content, microscopic anatomical status, DNA extraction concentration, and purity. For NGS testing reports, positive fusion results should include chromosome breakpoint positions, participation of tyrosine kinase structural domains, and in-frame fusion data. *NRG1/2* gene fusion involving the tyrosine kinase structural domain and being in-frame fusion should be reported as fusion; otherwise, it should be reported as rearrangement	Strongly recommended
	Consensus 9	When a physician has doubt, such as inconsistent results from different tests, new partner gene or fusion patterns, complex fusion events, unconfirmed involvement of fusion within the framework or full tyrosine kinase domain, and multiple driver gene positives, etc. discussing results and future treatment decisions with the Molecular Tumor Board (MTB) is strongly recommended	Strongly recommended
Treatment strategy	Consensus 10	For *NRG1/2* gene fusion solid tumors, if standard treatment fails, it is recommended to participation in related clinical trials for pan-ERBB TKIs such as afatinib and tarloxotinib, or ERBB2 inhibitory mAbs, ERBB3 inhibitory mAbs or dual anti-ERBB2/ERBB3 mAbs such as zenocutuzumab. For *NRG1/2* gene fusion tumor patients with drug resistance, performing NGS test to identify resistance mechanisms and deciding whether other related clinical trials are appropriate is recommended	Recommended

Abbreviations: mAb, monoclonal antibody; NGS, next-generation sequencing; TKI, tyrosine kinase inhibitor.

## Treatment Strategies for *NRG1/NRG2* Fusion

Currently, there are no approved targeted therapies specifically for the treatment of *NRG1* and *NRG2* fusions. However, several potential treatment strategies are being investigated in clinical trials. These include targeting *NRG1* fusion solid tumors using TKIs, monoclonal antibodies, or immunotherapy. Due to the intricate molecular pathways associated with *NRG1* fusion malignancies, novel therapeutic approaches that target specific mutations or signaling pathways have shown promise in preclinical studies and are currently being evaluated in clinical trials ( Table 2 ).

**Table 2:** Drugs under development for target *NRG1* fusion locally advanced or metastatic solid tumors (clinicaltrials.gov accessed on August 1, 2023)

Drug	Target	Developer	Study title	ClinicalTrials.gov ID	Cancer type	Phase	Status
Afatinib	Pan-ERBB TKIs	Boehringer Ingelheim	Afatinib in advanced *NRG1* -rearranged malignancies: the NCT/DKTK PMO-1604 phase-II trial	NCT04410653	Metastatic and locally advanced *NRG1* -rearranged malignancies	II	Active, not recruiting
			An open-labeled, single-arm clinical study to evaluate the efficacy of afatinib in treatment of locally advanced or metastatic non-small cell lung cancer with *NRG1* fusion	NCT04814056	*NRG1* -fused nonsmall cell lung cancer	IV	Not yet recruiting
Seribantumab	Anti-ERBB3 monoclonal antibody	Merrimack Pharmaceuticals	CRESTONE: a phase 2 study of seribantumab in adult patients with Neuregulin-1 ( *NRG1* ) fusion positive locally advanced or metastatic solid tumors	NCT04383210	Locally advanced or metastatic solid tumors	II	Active, not recruiting
			Single patient protocol for an *NRG1* fusion positive metastatic pancreatic cancer patient using seribantumab	NCT04790695	Metastatic pancreatic cancer	II	Completed
HMBD-001	Anti-ERBB3 monoclonal antibody	Hummingbird Bioscience	A phase 1b study to evaluate HMBD-001 with or without chemotherapy in participants with advanced solid tumors harboring *NRG1* gene fusions	NCT05919537	Nonsmall cell lung cancer Pancreatic cancer Locally advanced solid tumor Metastatic solid tumor	I/II	Not yet recruiting
Zenocutuzumab	Anti-ERBB2/ERBB3 monoclonal antibodies	Merus	A phase I/II study of MCLA-128, a full length IgG1 bispecific antibody targeting HER2 and HER3, in patients with solid tumors (eNRGy)	NCT02912949	Solid tumors harboring *NRG1* fusion	II	Recruiting
			Treatment plan of the HER2/HER3 bispecific antibody, MCLA-128, for a patient with advanced *NRG1* fusion positive solid tumor	NCT04100694	Solid tumor	I	Available
			A phase 2 study evaluating activity of zenocutuzumab (MCLA-128) in patients with or without molecularly defined cancers	NCT05588609	NSCLC harboring *NRG1* fusion Metastatic castration-resistant prostate cancer	II	Recruiting

Abbreviations: NSCLC, nonsmall cell lung cancer; TKI, tyrosine kinase inhibitor.

### Pan-ERBB Tyrosine Kinase Inhibitors

There are several clinical targeted approaches for the treatment of *NRG1* and *NRG2* fusion tumors, with the inhibition of the ERBB2–ERBB3 heterodimer activity being considered the most effective method.

### ERBB2 Selective Inhibitor

#### Afatinib

Afatinib, a pan-ERBB small molecule TKI, irreversibly inhibits tyrosine kinase autophosphorylation by binding to the kinase domains of EGFR, ERBB2, and ERBB4, leading to downregulation of the ERBB signaling. A case series report 46 included six cases of metastatic *NRG1* fusion tumors treated with afatinib, comprising five cases of metastatic lung cancer (two mucinous adenocarcinoma and three nonmucinous adenocarcinoma) and one case of metastatic colorectal cancer. Among these cases, one patient with IMA carrying *CD74* - *NRG1* fusion achieved partial remission for over 18 months after treatment with afatinib. Two patients with nonmucinous adenocarcinoma showed sustained responses for over 24 months. One patient with invasive lung mucinous adenocarcinoma carrying *SDC4* - *NRG1* fusion initially achieved partial remission for 5 months with afatinib (40 mg/d), but later experienced lung progression. After increasing the dose of afatinib to 50 mg/d, the patient achieved another 6 months of partial remission. Additionally, one patient with metastatic colorectal cancer carrying *POMK* - *NRG1* fusion and positive KRAS mutation achieved disease stability for 16 months with second-line treatment of afatinib. 46 An alliance composed of 22 centers from 9 countries in Europe, Asia, and the United States provided data on pathologically confirmed *NRG1* fusion lung cancer patients, showing an overall response rate (ORR) of 25% for afatinib, independent of the *NRG1* fusion subtype, and a median progression-free survival of 2.8 months. 37 Based on these study results, afatinib may be a treatment option for *NRG1* fusion tumors.

#### Tarloxotinib

Tarloxotinib is a prodrug that undergoes cleavage under hypoxic conditions to release an effective and irreversible pan-ERBB inhibitor. It represents a novel therapeutic approach that targets the tumor-specific hypoxic environment for cancer treatment. In the MDA-MB-175vIII breast cancer cell line harboring *DOC4* - *NRG1* fusion, tarloxotinib-E effectively inhibits the phosphorylation of ERBB2 and ERBB3 at concentrations similar to afatinib, while simultaneously suppressing the pERK1/2 and pAKT signals. 47 The Phase II RAIN-701 trial, which investigates the use of tarloxotinib as a monotherapy, includes a treatment arm targeting *NRG1* fusion tumors (NCT03805841). At present, the results of this subset have not been disclosed. 48

### ERBB3 Selective Inhibitor

#### Seribantumab (MM-121, FTN-001)

Seribantumab is a fully human anti-ERBB3 IgG _2_ monoclonal antibody. Preclinical experiments have shown that seribantumab inhibits the activation of ERBB3 signaling in cells carrying *NRG1* gene fusions and disrupts the stability of the entire ERBB family signaling pathway, including the activation of ERBB2, EGFR, and ERBB4. 49 Results from an ongoing Phase II clinical trial, CRESTONE (NCT04383210), evaluating the use of seribantumab in *NRG1* fusion-positive solid tumors, demonstrated an ORR of 33% across all cancer types, including two complete responses and a disease control rate of 92%. 50

#### Lumretuzumab

Lumretuzumab, a polyethylene glycol-engineered humanized monoclonal antibody developed by Roche, aims to inhibit the activation and signal transduction of ERBB3. 51 In cellular experiments using *SLC3A2* - *NRG1* fusion-positive HEK293T cells, lumretuzumab can inhibit the formation of ERBB2/ERBB3 heterocomplex induced by *SLC3A2* - *NRG1* fusion, thereby suppressing the activation of the PI3K/ERK/mTOR signaling pathway and the proliferation and growth of tumor cells. 52

### ERBB2/ERBB3 Selective Bispecific Monoclonal Antibodies

The ERBB2/ERBB3 bispecific monoclonal antibody, known as zenocutuzumab, targets both ERBB2 and ERBB3 receptors. By doing so, it effectively blocks the activation of ERBB3 by NRG1 fusion protein and inhibits the formation of heterodimers between ERBB2 and ERBB3. This mechanism of action has shown significant efficacy in patients with *NRG1* fusion.

### Zenocutuzumab (MCLA-128)

Zenocutuzumab is a bispecific human IgG _1_ antibody that contains two separate Fab arms specifically targeting the extracellular domains of ERBB2 and ERBB3. It can simultaneously inhibit the interaction between ERBB2 and NRG1, as well as the heterodimerization between ERBB3 and EGFR. This dual inhibition prevents ERBB3 and ERBB2 heterodimerization. 53 In a clinical trial involving *NRG1* fusion-positive/estrogen receptor-positive breast cancer patients who had experienced disease progression after treatment with cyclin-dependent kinase 4/6 inhibitors, zenocutuzumab demonstrated sustained tumor regression. 54 The I/II phase eNRGy clinical trial (NCT02912949) included patients with *NRG1* fusions in three cohorts: NSCLC (25 cases), pancreatic cancer (13 cases), and other solid tumors (13 cases). The results of the study showed excellent efficacy of zenocutuzumab in pancreatic cancer patients, with a partial response observed in 42% (5/12) of patients, stable disease in 6 cases, and disease progression in only 1 case. The objective response rate assessed by the researchers in pancreatic cancer was 40% (4/10). In three cases of chemotherapy-resistant *NRG1* fusion-positive pancreatic cancer patients, two patients experienced significant tumor shrinkage and sustained benefit for over 12 months: one patient with *ATP1B1* - *NRG1* gene fusion had a 44% reduction in tumor diameter at week 8 of treatment and a 54% reduction after 5 months of treatment, whereas another patient had a 22% reduction in tumor diameter at week 6 of treatment. In a case of *CD74* - *NRG1* -positive NSCLC patient who had previously received six systemic treatments including afatinib but experienced rapid disease progression, partial response was achieved for 7 months after switching to zenocutuzumab. 55 Targeting both ERBB2 and ERBB3 simultaneously with zenocutuzumab represents a new treatment approach for *NRG1* fusion-positive cancer patients. Based on this, in July 2020, the FDA granted orphan drug designation to zenocutuzumab for the treatment of *NRG1* fusion-positive pancreatic cancer patients.

### Drug Resistance

*NRG1* fusion has been identified as a potential mechanism of resistance to targeted therapies. For example, in breast cancer cell lines treated with lapatinib, increased expression of *NRG1* has been associated with acquired resistance to EGFR and ERBB2 kinase inhibitors. Overexpression of *NRG1* leads to reactivation of EGFR, ERBB2, and ERBB3 through phosphorylation. However, the combination of pertuzumab and lapatinib can inhibit *NRG1* -induced signaling more effectively than either drug alone. In animal models, this combination therapy has shown greater tumor regression compared with single-drug treatments. 56 Similarly, in selective inhibitors of nuclear export (SINE)-resistant ovarian cancer cell lines, the *NRG1* / *ERBB3* pathway is upregulated. The antitumor effect of SINE can be restored by removing ERBB3 using siRNA. 57 Additionally, exogenous *NRG1* can reduce the antitumor effect of SINE in ovarian cancer cell lines with high ERBB3 expression. In ALK-rearranged lung cancer, activation of the *NRG1* - *ERBB3* axis can cause resistance to lorlatinib. 58 However, pharmacological inhibition of ERBB3 or knockdown of the ERBB3 gene can restore sensitivity to lorlatinib in lung cancer cell lines. These findings suggest that targeting the *NRG1* / *ERBB3* axis may be a potential treatment option for resistant cancers. However, it is important to consider the ecological balance between ERBB receptors, as *NRG1* can bind to different receptors and unrestricted activation of other ligand–receptor axes may contribute to resistance. Therefore, future drug selection should aim to comprehensively inhibit the ERBB family signaling. 38

## Summary and Prospect

Tumor-driven fusion protein targets are highly valuable in targeted drug research. The significance of *NRG1* fusion in carcinogenesis was initially recognized in the mid-2010s, despite being first reported in breast cancer cell lines in 1997. The recent discovery of *NRG2* fusion further emphasizes its importance.

To detect fusion variants of *NRG1* and *NRG2* genes, particularly in their intronic regions, we propose RNA-based NGS technology, specifically WTS, as the optimal method. Comprehensive molecular profiling analysis of *NRG1* and *NRG2* fusion solid tumor patients can then identify potential therapeutic targets and guide personalized treatment strategies. This analysis can be achieved through NGS and other advanced genomic technologies. Alternatively, in cases where this is not feasible, IHC detection of pERBB3 levels can serve as a cost-effective preliminary screening method for *NRG1* fusion.

Understanding the molecular mechanisms and signaling pathways affecting *NRG1* and *NRG2* fusion genes is crucial for developing effective treatment strategies. Targeted therapies against these gene variants and signaling pathways have shown promising results in preclinical studies and early clinical trials. Drugs targeting the binding of NRG1 to ERBB3 and/or the heterodimerization of ERBB2/ERBB3, such as the bispecific monoclonal antibody zenocutuzumab, have demonstrated tumor volume reduction in *NRG1* fusion-positive tumors. These findings confirm that *NRG1* and *NRG2* gene fusions, although rare in solid tumors, are actionable oncogenic mutations. Patients who are *NRG1* positive and have failed standard treatment are recommended to participate in relevant clinical trials to increase their chances of benefiting.

In conclusion, the management of *NRG1* and *NRG2* fusion solid tumors necessitates a multidisciplinary approach that encompasses molecular detection methods, targeted therapies, and the selection of combination therapies. Further research and clinical trials are warranted to explore the most effective strategies for addressing these intricate malignancies.

## References

[1] Blume-Jensen P Hunter T Oncogenic kinase signalling Nature 2001 411 6835 355 365 11357143 10.1038/35077225

[2] Falls D L Neuregulins: functions, forms, and signaling strategies Exp Cell Res 2003 284 01 14 30 12648463 10.1016/s0014-4827(02)00102-7

[3] Jonna S Feldman R A Swensen J Detection of NRG1 gene fusions in solid tumors Clin Cancer Res 2019 25 16 4966 4972 30988082 10.1158/1078-0432.CCR-19-0160 PMC7470623

[4] Kohsaka S Hayashi T Nagano M Identification of novel CD74-NRG2α fusion from comprehensive profiling of lung adenocarcinoma in Japanese never or light smokers J Thorac Oncol 2020 15 06 948 961 32036070 10.1016/j.jtho.2020.01.021

[5] Ou S I Xiu J Nagasaka M Identification of novel *CDH1-NRG2* α and *F11R-NRG2α* fusions in NSCLC plus additional novel *NRG2α* fusions in other solid tumors by whole transcriptome sequencing JTO Clin Res Rep 2020 2 02 100132 34589990 10.1016/j.jtocrr.2020.100132 PMC8474258

[6] Odiete O Hill M F Sawyer D B Neuregulin in cardiovascular development and disease Circ Res 2012 111 10 1376 1385 23104879 10.1161/CIRCRESAHA.112.267286 PMC3752394

[7] Wilson K J Mill C P Cameron E M Hobbs S S Hammer R P Riese D J II Inter-conversion of neuregulin2 full and partial agonists for ErbB4 Biochem Biophys Res Commun 2007 364 02 351 357 17945187 10.1016/j.bbrc.2007.10.004 PMC2094732

[8] Hayes N V Gullick W J The neuregulin family of genes and their multiple splice variants in breast cancer J Mammary Gland Biol Neoplasia 2008 13 02 205 214 18415007 10.1007/s10911-008-9078-4

[9] Holmes W E Sliwkowski M X Akita R W Identification of heregulin, a specific activator of p185erbB2 Science 1992 256 5060 1205 1210 1350381 10.1126/science.256.5060.1205

[10] Wen D Peles E Cupples R Neu differentiation factor: a transmembrane glycoprotein containing an EGF domain and an immunoglobulin homology unit Cell 1992 69 03 559 572 1349853 10.1016/0092-8674(92)90456-m

[11] Marchionni M A Goodearl A D Chen M S Glial growth factors are alternatively spliced erbB2 ligands expressed in the nervous system Nature 1993 362 6418 312 318 8096067 10.1038/362312a0

[12] Falls D L Rosen K M Corfas G Lane W S Fischbach G D ARIA, a protein that stimulates acetylcholine receptor synthesis, is a member of the neu ligand family Cell 1993 72 05 801 815 8453670 10.1016/0092-8674(93)90407-h

[13] Tzahar E Levkowitz G Karunagaran D ErbB-3 and ErbB-4 function as the respective low and high affinity receptors of all Neu differentiation factor/heregulin isoforms J Biol Chem 1994 269 40 25226 25233 7929212

[14] Hanker A B Brown B P Meiler J Co-occurring gain-of-function mutations in HER2 and HER3 modulate HER2/HER3 activation, oncogenesis, and HER2 inhibitor sensitivity Cancer Cell 2021 39 08 1099 1.114E11 34171264 10.1016/j.ccell.2021.06.001 PMC8355076

[15] Olayioye M A Neve R M Lane H A Hynes N E The ErbB signaling network: receptor heterodimerization in development and cancer EMBO J 2000 19 13 3159 3167 10880430 10.1093/emboj/19.13.3159 PMC313958

[16] Busfield S J Michnick D A Chickering T W Characterization of a neuregulin-related gene, Don-1, that is highly expressed in restricted regions of the cerebellum and hippocampus Mol Cell Biol 1997 17 07 4007 4014 9199335 10.1128/mcb.17.7.4007 PMC232253

[17] Higashiyama S Horikawa M Yamada K A novel brain-derived member of the epidermal growth factor family that interacts with ErbB3 and ErbB4 J Biochem 1997 122 03 675 680 9348101 10.1093/oxfordjournals.jbchem.a021806

[18] Carraway K L III Weber J L Unger M J Neuregulin-2, a new ligand of ErbB3/ErbB4-receptor tyrosine kinases Nature 1997 387 6632 512 516 9168115 10.1038/387512a0

[19] Hobbs S S Coffing S L Le A T Neuregulin isoforms exhibit distinct patterns of ErbB family receptor activation Oncogene 2002 21 55 8442 8452 12466964 10.1038/sj.onc.1205960

[20] Breuleux M Role of heregulin in human cancer Cell Mol Life Sci 2007 64 18 2358 2377 17530167 10.1007/s00018-007-7120-0 PMC11138466

[21] Forster J A Paul A B Harnden P Knowles M A Expression of NRG1 and its receptors in human bladder cancer Br J Cancer 2011 104 07 1135 1143 21364580 10.1038/bjc.2011.39 PMC3068491

[22] Meyer D Birchmeier C Multiple essential functions of neuregulin in development Nature 1995 378 6555 386 390 7477375 10.1038/378386a0

[23] Schaefer G Fitzpatrick V D Sliwkowski M X Gamma-heregulin: a novel heregulin isoform that is an autocrine growth factor for the human breast cancer cell line, MDA-MB-175 Oncogene 1997 15 12 1385 1394 9333014 10.1038/sj.onc.1201317

[24] Fernandez-Cuesta L Thomas R K Molecular pathways: targeting NRG1 fusions in lung cancer Clin Cancer Res 2015 21 09 1989 1994 25501131 10.1158/1078-0432.CCR-14-0854

[25] Werr L Plenker D Dammert M A CD74-NRG1 fusions are oncogenic in vivo and induce therapeutically tractable ERBB2:ERBB3 heterodimerization Mol Cancer Ther 2022 21 05 821 830 35247925 10.1158/1535-7163.MCT-21-0820 PMC9377738

[26] Fernandez-Cuesta L Plenker D Osada H CD74-NRG1 fusions in lung adenocarcinoma Cancer Discov 2014 4 04 415 422 24469108 10.1158/2159-8290.CD-13-0633

[27] Adélaïde J Huang H E Murati A A recurrent chromosome translocation breakpoint in breast and pancreatic cancer cell lines targets the neuregulin/NRG1 gene Genes Chromosomes Cancer 2003 37 04 333 345 12800145 10.1002/gcc.10218

[28] Huang H E Chin S F Ginestier C A recurrent chromosome breakpoint in breast cancer at the NRG1/neuregulin 1/heregulin gene Cancer Res 2004 64 19 6840 6844 15466169 10.1158/0008-5472.CAN-04-1762

[29] Duruisseaux M McLeer-Florin A Antoine M NRG1 fusion in a French cohort of invasive mucinous lung adenocarcinoma Cancer Med 2016 5 12 3579 3585 27770508 10.1002/cam4.838 PMC5224837

[30] Jung Y Yong S Kim P VAMP2-NRG1 fusion gene is a novel oncogenic driver of non-small-cell lung adenocarcinoma J Thorac Oncol 2015 10 07 1107 1111 26134228 10.1097/JTO.0000000000000544

[31] Jones M R Williamson L M Topham J T *NRG1* gene fusions are recurrent, clinically actionable gene rearrangements in *KRAS* wild-type pancreatic ductal adenocarcinoma Clin Cancer Res 2019 25 15 4674 4681 31068372 10.1158/1078-0432.CCR-19-0191

[32] Heining C Horak P Uhrig S *NRG1* fusions in *KRAS* wild-type pancreatic cancer Cancer Discov 2018 8 09 1087 1095 29802158 10.1158/2159-8290.CD-18-0036

[33] Jones J T Akita R W Sliwkowski M X Binding specificities and affinities of EGF domains for ErbB receptors FEBS Lett 1999 447(2-3):227 231 10214951 10.1016/s0014-5793(99)00283-5

[34] Cha Y J Lee C Joo B Kim K A Lee C K Shim H S Clinicopathological characteristics of NRG1 fusion-positive solid tumors in Korean patients Cancer Res Treat 2023 55 04 1087 1095 37321274 10.4143/crt.2023.682 PMC10582527

[35] Yuan H CS Wang L Dong X Wang A Wang K The landscape of NRG1 fusions based on NGS in Chinese solid tumor patients ASCO 2022 x e15073

[36] Nagasaka M Ou S I Neuregulin 1 fusion-positive NSCLC J Thorac Oncol 2019 14 08 1354 1359 31128291 10.1016/j.jtho.2019.05.015

[37] Drilon A Duruisseaux M Han J Y Clinicopathologic features and response to therapy of *NRG1* fusion-driven lung cancers: the eNRGy1 Global Multicenter Registry J Clin Oncol 2021 39 25 2791 2802 34077268 10.1200/JCO.20.03307 PMC8407651

[38] Nagasaka M Ou S I NRG1 and NRG2 fusion positive solid tumor malignancies: a paradigm of ligand-fusion oncogenesis Trends Cancer 2022 8 03 242 258 34996744 10.1016/j.trecan.2021.11.003

[39] Trombetta D Graziano P Scarpa A Frequent *NRG1* fusions in Caucasian pulmonary mucinous adenocarcinoma predicted by phospho-ErbB3 expression Oncotarget 2018 9 11 9661 9671 29515761 10.18632/oncotarget.23800 PMC5839392

[40] Jones M R Lim H Shen Y Successful targeting of the NRG1 pathway indicates novel treatment strategy for metastatic cancer Ann Oncol 2017 28 12 3092 3097 28950338 10.1093/annonc/mdx523

[41] Howarth K D Mirza T Cooke S L NRG1 fusions in breast cancer Breast Cancer Res 2021 23 01 3 33413557 10.1186/s13058-020-01377-5 PMC7788813

[42] Mercer T R Gerhardt D J Dinger M E Targeted RNA sequencing reveals the deep complexity of the human transcriptome Nat Biotechnol 2011 30 01 99 104 22081020 10.1038/nbt.2024 PMC3710462

[43] Song Z Xu C He Y Simultaneous detection of gene fusions and base mutations in cancer tissue biopsies by sequencing dual nucleic acid templates in unified reaction Clin Chem 2020 66 01 178 187 31810998 10.1373/clinchem.2019.308833

[44] Zheng Z Liebers M Zhelyazkova B Anchored multiplex PCR for targeted next-generation sequencing Nat Med 2014 20 12 1479 1484 25384085 10.1038/nm.3729

[45] Lanic M D Le Loarer F Rainville V Detection of sarcoma fusions by a next-generation sequencing based-ligation-dependent multiplex RT-PCR assay Mod Pathol 2022 35 05 649 663 35075283 10.1038/s41379-021-00980-x

[46] Cadranel J Liu S V Duruisseaux M Therapeutic potential of afatinib in NRG1 fusion-driven solid tumors: a case series Oncologist 2021 26 01 7 16 32852072 10.1634/theoncologist.2020-0379 PMC7794194

[47] Estrada-Bernal A Le A T Doak A E Tarloxotinib is a hypoxia-activated Pan-HER kinase inhibitor active against a broad range of HER-family oncogenes Clin Cancer Res 2021 27 05 1463 1475 33355298 10.1158/1078-0432.CCR-20-3555 PMC7926264

[48] Liu S V NRG1 fusions: biology to therapy Lung Cancer 2021 158 25 28 34098222 10.1016/j.lungcan.2021.05.011

[49] Odintsov I Lui A JW Sisso W J The anti-HER3 mAb seribantumab effectively inhibits growth of patient-derived and isogenic cell line and xenograft models with oncogenic *NRG1* fusions Clin Cancer Res 2021 27 11 3154 3166 33824166 10.1158/1078-0432.CCR-20-3605 PMC8172458

[50] Thavaneswaran S Chan W Y Asghari R Clinical response to seribantumab, an anti-human epidermal growth factor receptor-3 immunoglobulin 2 monoclonal antibody, in a patient with metastatic pancreatic ductal adenocarcinoma harboring an *NRG1* fusion JCO Precis Oncol 2022 6 e2200263 36455193 10.1200/PO.22.00263 PMC9812631

[51] Meneses-Lorente G Friess T Kolm I Preclinical pharmacokinetics, pharmacodynamics, and efficacy of RG7116: a novel humanized, glycoengineered anti-HER3 antibody Cancer Chemother Pharmacol 2015 75 04 837 850 25702049 10.1007/s00280-015-2697-8 PMC4365277

[52] Shin D H Jo J Y Han J Y Dual targeting of ERBB2/ERBB3 for the treatment of SLC3A2-NRG1-mediated lung cancer Mol Cancer Ther 2018 17 09 2024 2033 29959202 10.1158/1535-7163.MCT-17-1178

[53] Geuijen C AW De Nardis C Maussang D Unbiased combinatorial screening identifies a bispecific IgG1 that potently inhibits HER3 signaling via HER2-guided ligand blockade Cancer Cell 2021 39 08 1163 1164 34375611 10.1016/j.ccell.2021.07.015

[54] Fontana E Torga G Fostea R Sustained tumor regression with zenocutuzumab, a bispecific antibody targeting human epidermal growth factor receptor 2/human epidermal growth factor receptor 3 signaling, in *NRG1* fusion-positive, estrogen receptor-positive breast cancer after progression on a cyclin-dependent kinase 4/6 inhibitor JCO Precis Oncol 2022 6 e2100446 35977350 10.1200/PO.21.00446

[55] Schram A M Odintsov I Espinosa-Cotton M Zenocutuzumab, a HER2xHER3 bispecific antibody, is effective therapy for tumors driven by NRG1 gene rearrangements Cancer Discov 2022 12 05 1233 1247 35135829 10.1158/2159-8290.CD-21-1119 PMC9394398

[56] Leung W Y Roxanis I Sheldon H Combining lapatinib and pertuzumab to overcome lapatinib resistance due to NRG1-mediated signalling in HER2-amplified breast cancer Oncotarget 2015 6 08 5678 5694 25691057 10.18632/oncotarget.3296 PMC4467394

[57] Miyake T M Pradeep S Bayraktar E NRG1/ERBB3 pathway activation induces acquired resistance to XPO1 inhibitors Mol Cancer Ther 2020 19 08 1727 1735 32499298 10.1158/1535-7163.MCT-19-0977 PMC7415525

[58] Taniguchi H Akagi K Dotsu Y Pan-HER inhibitors overcome lorlatinib resistance caused by NRG1/HER3 activation in ALK-rearranged lung cancer Cancer Sci 2023 114 01 164 173 36086904 10.1111/cas.15579 PMC9807501

